# Higher Appendicular Skeletal Muscle Mass Protects Metabolically Healthy Obese Boys but Not Girls from Cardiometabolic Abnormality

**DOI:** 10.3390/ijerph16040652

**Published:** 2019-02-22

**Authors:** Seung-Nam Kim, Jaehee Kim

**Affiliations:** 1 College of Korean Medicine, Dongguk University, Dongguk-ro, Ilsandong-gu, Goyang-si, Gyeonggi-do 10326, Korea; sk2013ny@gmail.com; 2 Graduate School of Alternative Medicine, Kyonggi University, 63, Kyonggidae-ro 9-gil, Seodaemun-gu, Seoul 120-837, Korea

**Keywords:** obesity, skeletal muscle mass, adolescent, gender difference, cardiometabolic abnormalities

## Abstract

Factors related to metabolically healthy obesity (MHO) are not well characterized in adolescents. The study’s aim was to investigate the impact of skeletal muscle mass (SMM) on MHO in adolescents. A secondary analysis was performed using the data of 221 Korean overweight and obese adolescents aged 12–18 years from the Korean National Health and Nutrition Examination Survey. Appendicular skeletal muscle (ASM) mass and total body fat mass were measured by dual-energy X-ray absorptiometry. Being metabolically unhealthy was defined using three definitions: Having ≥1, ≥2, or ≥3 cardiometabolic risk factors (CRFs; waist circumference, blood pressure, glucose, triglycerides, and HDL-cholesterol). Multiple logistic regression analyses adjusted for age and lifestyle factors were performed to assess the association between ASM and MHO. In boys, the risk for having either ≥2 CRFs or ≥3 CRFs was significantly lower with higher weight-adjusted ASM and ratio of ASM to fat mass after controlling for covariates, but this association was not significant with CRFs ≥ 1. In girls, all adjusted odds ratios were not significant. Findings indicate that SMM is a potentially protective factor against cardiometabolic abnormality in adolescents with MHO, showing gender difference. This heightens the importance of SMM in the management of obesity, especially in boys.

## 1. Introduction

Obesity is commonly associated with cardiometabolic abnormality but coexists with a healthy metabolic profile [1]. This metabolically healthy obese (MHO) phenotype has also been identified in adolescents [2]. Generally, factors related to MHO phenotype are not well characterized [1]. A recent finding in obese youth showed that MHO adolescents were less sedentary and more engaged in moderate-to-vigorous activity compared to metabolically unhealthy obese (MUO) adolescents with no differences between two phenotypes for cardiorespiratory fitness and muscle strength [2]. Another plausible protective factor for preserving cardiometabolic health in MHO adolescents is skeletal muscle mass (SMM) since, in adults, SMM is inversely associated with cardiometabolic abnormalities [3,4]. Skeletal muscle is known to be responsible for regulation of lipid metabolism and whole-body glucose uptake [5,6]. Therefore, we speculated that higher skeletal muscle mass relative to body weight or fat mass might play a role in reducing the risk of cardiometabolic abnormality in obese adolescents. So far, the potential role of SMM on metabolically healthy phenotype in overweight or obese adolescents is unknown.

A favorable effect of lean mass including SMM on metabolic profiles might be overlooked in the obese population since obese children and adolescents have higher lean mass than those with normal weight [7]. However, interestingly obese youths have lower lean mass to fat mass (FM) ratio than those with normal weight after controlling for age, height, and pubertal stage in both genders [7]. This implies that relative amount of SMM or its ratio of FM could explain, at least in part, the metabolic difference observed between two obesity phenotypes. Therefore, we examined a relationship between SMM and cardiometabolic abnormality after controlling for age and lifestyle factors in overweight and obese adolescents. We hypothesized that obese adolescents with more SMM might have a lower risk of being MUO. In addition, the MHO phenotype has been classified on the basis of the different definitions, resulting in a wide range of prevalence [1,8]. There are no consistent criteria for the definition of MHO phenotype as either free of or having fewer of cardiometabolic risk factors (CRFs) has been used to assess it [1,8]. Likewise, we examined our hypothesis using three different definitions of MHO phenotype defined by the number of CRFs.

## 2. Materials and Methods

### 2.1. Study Population

We performed a secondary analysis using the data of 221 overweight and obese boys (N = 110) and girls (N = 111) aged 12 to 18 years from a nationally representative cross-sectional sample of the civilian non-institutionalized population. We used the data from the Korean National Health and Nutrition Examination Survey (KNHANES), conducted by the Korea Centers for Disease Control and Prevention (KCDC). Details of the KNHANES design, procedures, and participants have been published elsewhere [9]. In brief, the KNHANES were conducted annually using a rolling sampling design. Sampling units were defined based on geographic area, gender, and age group using the household registries from the National Census Registry in South Korea.

We analyzed the open data from the 2009–2011 KNHANES, which included whole body composition measures by dual-energy X-ray absorptiometry (DXA). Among 2372 participants aged 12 to 18 years, those without weight and height measurements, being underweight or normal weight, and diagnosed with congenital heart disease, seizures, any type of cancer, and developmental disabilities were excluded. Among 419 overweight and obese adolescents, participants who had missing data for dietary intake, physical activity, waist circumference, blood pressure and blood variables, or DXA measurements, as well as those who fasted less than 8 h, were further excluded from the analysis. The final sample included 221 adolescents. The protocol of KNHANES was approved by the KCDC’s Institutional Review Boards. All participants gave written informed consent. 

### 2.2. Definition of Metabolically Healthy Obesity

Overweight and obesity were defined as body mass index (BMI (kg/m^2^)) ≥ 85th percentile value for age and sex based on the Standard Growth Charts of the Korean Pediatric Society. Being MUO was defined as having ≥1, ≥2, or ≥3 CRFs. CRFs were determined by 5 diagnostic criteria for metabolic syndrome using age-modified guidelines of the National Cholesterol Education Program (NCEP) Adult Treatment Panel (ATP) III: (1) systolic or diastolic blood pressure ≥90th percentile value for sex, age, and height percentile values or on antihypertensive drug treatment (those 18 years old: ≥130/85 mmHg); (2) fasting plasma glucose ≥100 mg/dL or on antidiabetic drug treatment; (3) triglyceride concentration ≥110 mg/dL; (4) HDL cholesterol concentration ≤40 mg/dL; (5) waist circumference ≥90th percentile value for sex and age [10]. Three definitions were used to define MHO phenotype: (1) having no CRFs; (2) having 0 or 1 CRF; (3) having 0 to 2 CRFs.

### 2.3. Skeletal Muscle Mass and Fat Mass

Appendicular lean soft tissue (ALST) and FM were measured by DXA (Hologic, Bedford, MA, USA). ALST is known to be well correlated with total-body SMM measured by whole-body magnetic resonance imaging [11]. Appendicular skeletal muscle (ASM) was calculated as the sum of ALST, and then normalized for body weight ([ASM/weight] × 100). The ASM to FM ratio was calculated as ASM divided by total body FM. 

### 2.4. Physical Activity and Dietary Intake

The self-reported physical activity questionnaire was used to determine if participants met the recommended levels of physical activity by the Physical Activity Guidelines for American children and adolescents (moderate-to-vigorous physical activity ≥60 min per day) [12]. Participants were classified into two groups by whether they met the physical activity recommendations or not. Dietary factors include carbohydrate intake (≥60% of total energy intake vs. <60%) and fat intake (<30% of total energy intake vs. ≥30%).

### 2.5. Statistical Analysis

The KNHANES data was collected through complex sample design using a stratified, multistage, probability sampling design [9]. Due to the complex sampling design of KHANES, sample weights were applied to make the studied sample representative of the Korean adolescent population aged 12 to 18 years [13]. Accordingly, our sample represented approximately 253,060 boys and 264,685 girls. The data was analyzed using the SPSS complex samples procedure (SPSS version 22.0, IBM SPSS Inc., Chicago, IL, USA) with a significance level of *p* < 0.05.

Significance of difference in prevalence of MHO between boys and girls was tested by chi-square test with complex samples crosstabs. Difference in body composition between the MHO and MUO groups was tested by general linear model for complex sample design. To assess the association between muscle variables and risks for being MUO, multiple logistic regression analysis for complex samples was performed with adjustment for age (12–15 years vs. 16–18 years), fat intake, carbohydrate intake, and moderate and vigorous physical activity. Adjusted odds ratios (ORs) of muscle variables for being MUO were estimated with 95% confidence interval (CI). In the multiple logistic regression models for complex samples, the muscle variables were continuous variables with 1-percent unit increase for the weight-adjusted ASM and 0.1 unit increase for the ASM to FM ratio. The interpretation of the muscle variables in the model is that for every 1 unit of increase in muscle variables, the odds of being MUO decrease (or increase), adjusting for covariates [14].

## 3. Results

The prevalence of MHO phenotype is presented in Table 1 by sex and three definitions. Using the first definition of MHO phenotype (0 CRFs), there was no significant difference of the MHO prevalence between male and female adolescents. Using the second definition (0 or 1 CRFs), the MHO prevalence of boys tended to be lower than that of girls (*p* = 0.08). Using the third definition (0–2 CRFs), the percentage of MHO boys was significantly lower than that of girls (*p* < 0.01).

The anthropometric characteristics and body composition of the MHO and MUO adolescents are presented in Table 1 by sex and three definitions. Using the first definition of MHO phenotype, there were no significant differences in the weight-adjusted ASM, ASM to FM ratio, and body fat percentage between the MHO and MUO boys. According to the second definition, the differences of the weight-adjusted ASM and ASM to FM ratio between two groups tended to get significant (both *p* = 0.06). Using the third definition, both weight-adjusted ASM and ASM to FM ratio were significantly higher in MHO boys compared to MUO boys (both *p* < 0.05). Body fat percentage tended to be lower in MHO boys compared to MUO boys (*p* = 0.05).

In girls, no significant differences in the weight-adjusted ASM, ASM to FM ratio, and body fat percentage were observed between two groups regardless of definitions of MHO phenotype.

In male adolescents, the adjusted ORs of being MUO for the weight-adjusted ASM and the ratio of ASM to FM were not significant when MUO phenotype was defined by having ≥1 CRFs (model 1). However, when MUO phenotype was defined by having either ≥2 CRFs (model 2) or ≥3 CRFs (model 3), the risk of being MUO was significantly lower with higher weight-adjusted ASM and the ratio of ASM to FM after controlling for age, fat intake, carbohydrate intake, and physical activity (*p* < 0.01 for model 2 and *p* < 0.05 for model 3) (Table 2). In female adolescents, all adjusted odds ratios were not significant, suggesting that there is no association between being MUO and SMM.

## 4. Discussion

Our results showed that the risk of being metabolically unhealthy is lower with higher muscle mass relative to weight or fat mass in overweight and obese boys but not in overweight and obese girls, suggesting that SMM may be a potentially protective factor against cardiometabolic abnormality in MHO adolescents. These findings seemed to depend on definition of MHO phenotype showing gender differences. 

Since obese individuals have greater lean mass including SMM along with more fat mass compared to the lean counterparts [7], much attention has focused on clinical implications of excess body fat mass rather than muscle mass in overweight and obese individuals [15]. However, inter-individual variability in body composition (i.e., fat distribution, relative amount of skeletal muscle mass) exists in obese individuals with the same BMI [16]. A greater understanding of the factors related to MHO phenotype is important for therapeutic decision-making because the most appropriate care for the MHO individuals is not well established [1]. Our findings may increase awareness of the need for assessing muscle mass in obese adolescents. 

One plausible reason for gender differences observed in the present findings is that other factors related to cardiometabolic abnormality could confound an association of muscle mass with being MHO: Visceral obesity is independently associated with cardiometabolic abnormality in obese adolescents [17]. In female obese adolescents, visceral adipose tissue might be one of plausible candidates related to being MUO.

Relatively favorable clinical implications of MHO phenotypes and some possibilities of the future development of CRFs have been controversial [18,19]. In adults, one recent study showed that hazard ratio for incidence of cardiovascular disease did not increase over a 12-year follow-up in MHO individuals compared to those with metabolically healthy normal weight (MHNW) [18]. In contrast, another recent study found that more MHO adults developed one or more cardiometabolic risk factor or complications than MHNW individuals over an average 16-year follow-up, especially with weight gain [19]. In children and adolescents, the long-term clinical implications of the MHO phenotype are unknown.

Despite poorly understood mechanisms and ambiguity about long-term effects associated with MHO status, some previous studies have proposed that identification of factors related to this phenotype instead of weight loss per se would be helpful for making therapeutic decisions and in medical education to delay or protect these obese individuals from metabolic abnormalities [20,21,22]. Although findings are conflicting [22], being less sedentary, participating in more moderate-to-vigorous physical activity, and eating more fruits and vegetables were reported to be associated with MHO status [2,21,23,24]. Regarding contribution to understanding the MHO phenotype, our findings indicate that having a higher SMM is associated with cardiometabolic health in obese male adolescents. Similar to our finding from overweight and obese boys, it has been recently reported that low ASM increases the risks of having cardiometabolic diseases in overweight and obese men [25]. Our study sample included overweight adolescents (85th ≤ BMI < 95th percentile value for age and sex). Therefore, our findings may need to be confirmed in the pure obese adolescent population (BMI ≥ 95th percentile value).

One possible weakness of the present study may be that the study population was limited to Asian overweight and obese adolescents. Another limitation of the present study is that pubertal stages were not accounted for. In the future studies, our findings need to be confirmed in other race/ethnic groups with assessment of pubertal status. Our study was cross-sectional and thus was not able to determine a causal relationship between SMM and being MHO. We used the metabolic syndrome criteria to define cardiometabolic health. MHO adults have other cardiometabolic abnormalities such as higher insulin, insulin resistance, and C-reactive protein levels than individuals with normal weight [26]. Therefore, the association of other cardiometabolic factors with SMM should be studied further.

## 5. Conclusions

Our findings indicate that the risk of being metabolically unhealthy is lower with higher muscle mass in overweight and obese boys but not in overweight and obese girls, suggesting that muscle mass may be a potentially protective factor against cardiometabolic abnormality in MHO boys. This heightens the importance of the idea that the assessment of muscle mass in this population is clinically important in the management of obesity.

## Figures and Tables

**Table 1:** Prevalence, characteristics, and body composition of MHO.

		**Boys (N = 110)**			**Girls (N = 111)**		
**Definition of MUO**		**MHO**	**MUO**		**MHO**	**MUO**	***p* Value ^b^**
(1) ≥1 component of metabolic syndrome		15.9%	84.1%		19.7%	80.3%	0.532
(2) ≥2 components of metabolic syndrome		43.6%	56.4%		56.4%	43.6%	0.081
(3) ≥3 components of metabolic syndrome		69.4%	30.6%		88.8%	11.2%	0.003
		**Boys (N = 110)**			**Girls (N = 111)**		
	**Variables ^a^**	**MHO**	**MUO**	***p* Value**	**MHO**	**MUO**	***p* Value ^c^**
First definition	N	20	90		23	88	
	Age (y)	14.83 ± 0.68	14.80 ± 0.20		14.94 ± 0.52	15.00 ± 0.26	
	Height (cm)	171.38 ± 2.49	171.51 ± 0.95		158.04 ± 1.15	160.51 ± 0.84	
	Weight (kg)	76.13 ± 3.06	80.08 ± 1.37		61.38 ± 0.67	68.56 ± 1.05	
	ASM/weight (%)	30.58 ± 0.74	30.04 ± 0.27	0.497	24.87 ± 0.71	24.32 ± 0.39	0.497
	Body fat (%)	30.13 ± 1.50	31.36 ± 0.55	0.441	37.63 ± 1.36	39.39 ± 0.73	0.233
	ASM/total fat	1.07 ± 0.08	1.02 ± 0.03	0.544	0.69 ± 0.04	0.64 ± 0.02	0.349
Second definition	N	51	59		66	45	
	Age (y)	14.52 ± 0.35	15.03 ± 0.23		14.84 ± 0.23	15.17 ± 0.36	
	Height (cm)	169.09 ± 1.47	173.34 ± 0.84		159.26 ± 0.79	161.02 ± 0.79	
	Weight (kg)	73.85 ± 1.75	83.79 ± 1.30		64.65 ± 0.88	70.37 ± 1.48	
	ASM/weight (%)	30.73 ± 0.45	29.66 ± 0.29	0.059	24.75 ± 0.44	24.01 ± 0.54	0.286
	Body fat (%)	30.16 ± 0.94	31.95 ± 0.59	0.111	38.26 ± 0.88	40.06 ± 0.93	0.137
	ASM/total fat	1.10 ± 0.06	0.97 ± 0.03	0.057	0.68 ± 0.03	0.62 ± 0.03	0.132
Third definition	N	78	32		99	12	
	Age (y)	14.60 ± 0.28	15.27 ± 0.25		14.94 ± 0.20	15.36 ± 0.70	
	Height (cm)	169.87 ± 1.25	175.16 ± 0.81		160.16 ± 0.68	158.97 ± 1.32	
	Weight (kg)	75.34 ± 1.40	88.78 ± 1.71		66.34 ± 0.98	73.55 ± 1.83	
	ASM/weight (%)	30.46 ± 0.33	29.36 ± 0.10	0.042	24.44 ± 0.36	24.33 ± 0.99	0.909
	Body fat (%)	30.57 ± 0.70	32.52 ± 0.67	0.050	38.89 ± 0.69	40.21 ± 1.59	0.406
	ASM/total fat	1.06 ± 0.04	0.94 ± 0.04	0.035	0.66 ± 0.02	0.63 ± 0.05	0.637

ASM, appendicular skeletal muscle mass; MHO, metabolically healthy overweight/obese individuals; MUO, metabolically unhealthy overweight/obese individuals. ^a^ Data is presented in estimated mean ± standard error. ^b^ Significance of difference in prevalence between boys and girls was tested by chi-square test with complex samples crosstabs. ^c^ Significance of difference in body composition between MHO and MUO was tested by general linear model for complex sample design.

**Table 2:** Association between skeletal muscle mass and risk of being metabolically unhealthy in overweight/obese adolescents.

		Boys (N = 110)	*p* Value	Girls (N = 110)	*p* Value
Adjusted odds ratios for cardiometabolic abnormality (95% Confidence interval)					
ASM/weight (%)	Model 1 (≥ 1)	0.94 (0.77–1.14)	0.523	0.95 (0.74–1.21)	0.663
	Model 2 (≥ 2)	0.79 (0.66–0.94)	0.007	0.92 (0.76–1.12)	0.405
	Model 3 (≥ 3)	0.78 (0.64–0.95)	0.014	1.07 (0.84–1.38)	0.579
ASM/total fat	Model 1 (≥ 1)	0.95 (0.82–1.11)	0.534	0.84 (0.53–1.35)	0.473
	Model 2 (≥ 2)	0.80 (0.69–0.93)	0.004	0.79 (0.56–1.10)	0.159
	Model 3 (≥ 3)	0.79 (0.66–0.96)	0.015	1.05 (0.70–1.57)	0.818

ASM, appendicular skeletal muscle mass. All odds ratios are adjusted for age, fat intake, carbohydrate intake, and moderate and vigorous physical activity. Model 1: Being metabolically unhealthy was defined by having one or more component of metabolic syndrome. Model 2: Being metabolically unhealthy was defined by having two or more components of metabolic syndrome. Model 3: Being metabolically unhealthy was defined by having three or more components of metabolic syndrome.

## References

[1] Samocha-Bonet D., Dixit V.D., Kahn C.R., Leibel R.L., Lin X., Nieuwdorp M., Pietiläinen K.H., Rabasa-Lhoret R., Roden M., Scherer P.E. (2014). Metabolically healthy and unhealthy obese—The 2013 stock conference report. Obes. Rev..

[2] Cadenas-Sanchez C., Ruiz J.R., Labayen I., Huybrechts I., Manios Y., González-Gross M., Breidenassel C., Kafatos A., De Henauw S., Vanhelst J. (2017). Prevalence of metabolically healthy but overweight/obese phenotype and its association with sedentary time, physical activity, and fitness. J. Adolesc. Health.

[3] Atlantis E., Martin S.A., Haren M.T., Taylor A.W., Wittert G.A. (2009). Members of the florey adelaide male ageing study. Inverse associations between muscle mass, strength, and the metabolic syndrome. Metabolism.

[4] Srikanthan P., Karlamangla A.S. (2011). Relative muscle mass is inversely associated with insulin resistance and prediabetes. Findings from the third National Health and Nutrition Examination Survey. J. Clin. Endocrinol. Metab..

[5] Jacob S., Hauer B., Becker R., Artzner S., Grauer P., Löblein K., Nielsen M., Renn W., Rett K., Wahl H.G. (1999). Lipolysis in skeletal muscle is rapidly regulated by low physiological doses of insulin. Diabetologia.

[6] Baron A.D., Brechtel G., Wallace P., Edelman S.V. (1988). Rates and tissue sites of non-insulin- and insulin-mediated glucose uptake in humans. Am. J. Physiol..

[7] Garg M.K., Marwaha R.K., Mahalle N., Tandon N. (2016). Relationship of lean mass and obesity in Indian urban children and adolescents. Indian J. Endocrinol. Metab..

[8] Phillips C.M., Dillon C., Harrington J.M., Mc Carthy V.J., Kearney P.M., Fitzgerald A.P., Perry I.J. (2013). Defining metabolically healthy obesity: Role of dietary and lifestyle factors. PLoS ONE.

[9] Kweon S., Kim Y., Jang M.J., Kim K., Choi S., Chun C., Khang Y.H., Oh K. (2014). Data resource profile: The Korea National Health and Nutrition Examination Survey (KNHANES). Int. J. Epidemiol..

[10] Ford E.S., Li C. (2008). Defining the metabolic syndrome in children and adolescents: Will the real definition please stand up?. J. Pediatr..

[11] Kim J., Shen W., Gallagher D., Jones A., Wang Z., Wang J., Heshka S., Heymsfield S.B. (2006). Total-body skeletal muscle mass: Estimation by dual-energy X-ray absorptiometry in children and adolescents. Am. J. Clin. Nutr..

[12] U.S. Department of Health and Human Services Physical Activity Guidelines for Americans.

[13] Saylor J., Friedmann E., Lee H.J. (2012). Navigating complex sample analysis using national survey data. Nurs. Res..

[14] Ranganathan P., Pramesh C.S., Aggarwal R. (2017). Common pitfalls in statistical analysis: Logistic regression. Perspect Clin Res..

[15] Bastien M., Poirier P., Lemieux I., Després J.P. (2014). Overview of epidemiology and contribution of obesity to cardiovascular disease. Prog. Cardiovasc. Dis..

[16] Müller M.J., Lagerpusch M., Enderle J., Schautz B., Heller M., Bosy-Westphal A. (2012). Beyond the body mass index: Tracking body composition in the pathogenesis of obesity and the metabolic syndrome. Obes. Rev..

[17] Weiss R., Dufour S., Taksali S.E., Tamborlane W.V., Petersen K.F., Bonadonna R.C., Boselli L., Barbetta G., Allen K., Rife F. (2003). Prediabetes in obese youth: A syndrome of impaired glucose tolerance, severe insulin resistance, and altered myocellular and abdominal fat partitioning. Lancet.

[18] Mirzaei B., Abdi H., Serahati S., Barzin M., Niroomand M., Azizi F., Hosseinpanah F. (2017). Cardiovascular risk in different obesity phenotypes over a decade follow-up: Tehran lipid and glucose study. Atherosclerosis.

[19] De Ycaza A.E., Donegan D., Jensen M.D. (2018). Long-term metabolic risk for the metabolically healthy overweight/obese phenotype. Int. J. Obes..

[20] Primeau V., Coderre L., Karelis A.D., Brochu M., Lavoie M.E., Messier V., Sladek R., Rabasa-Lhoret R. (2011). Characterizing the profile of obese patients who are metabolically healthy. Int. J. Obes..

[21] Roberge J.B., Van Hulst A., Barnett T.A., Drapeau V., Benedetti A., Tremblay A., Henderson M. (2019). Lifestyle habits, dietary factors, and the metabolically unhealthy obese phenotype in youth. J. Pediatr..

[22] Heinzle S., Ball G.D., Kuk J.L. (2015). Variations in the prevalence and predictors of prevalent metabolically healthy obesity in adolescents. Pediatr. Obes..

[23] Kuzik N., Carson V., Andersen L.B., Sardinha L.B., Grontved A., Hansen B.H., Ekelund U., International Children’s Accelerometry Database (ICAD) Collaborators (2017). Physical activity and sedentary time associations with metabolic health across weight statuses in children and adolescents. Obesity.

[24] Prince R.L., Kuk J.L., Ambler K.A., Dhaliwal J., Ball G.D. (2014). Predictors of metabolically healthy obesity in children. Diabetes Care.

[25] Khazem S., Itani L., Kreidieh D., El Masri D., Tannir H., Citarella R., El Ghoch M. (2018). Reduced lean body mass and cardiometabolic diseases in adult males with overweight and obesity: A pilot study. Int. J. Environ. Res. Public Health.

[26] Manu P., Ionescu-Tirgoviste C., Tsang J., Napolitano B.A., Lesser M.L., Correll C.U. (2012). Dysmetabolic signals in “metabolically healthy” obesity. Obes. Res. Clin. Pract..

